# CAN THE SIZE-SPECIFIC DOSE ESTIMATE BE DERIVED FROM THE BODY MASS INDEX? A FEASIBILITY STUDY

**DOI:** 10.1093/rpd/ncac038

**Published:** 2022-04-21

**Authors:** Beatrice Steiniger, Chris Klippel, Ulf Teichgräber, Jürgen R Reichenbach, Martin Fiebich

**Affiliations:** Department of Diagnostic and Interventional Radiology, University Hospital Jena, Am Klinikum 1, Jena 07747, Germany; Department of Diagnostic and Interventional Radiology, University Hospital Jena, Am Klinikum 1, Jena 07747, Germany; Department of Diagnostic and Interventional Radiology, University Hospital Jena, Am Klinikum 1, Jena 07747, Germany; Medical Physics Group, Department of Diagnostic and Interventional Radiology, University Hospital Jena, Philosophenweg 3, Jena 07743, Germany; Institute of Medical Physics and Radiation Protection, University of Applied Sciences Giessen, Wiesenstraße 14, Gießen 35390, Germany

## Abstract

Size-specific dose estimate ($\mathbf{SSDE}$) index appears to be more suitable than the commonly used volume computed tomography dose index ($\mathbf{C}{\mathbf{TDI}}_{\mathbf{vol}}$) to estimate the dose delivered to the patient during a computed tomography (CT) scan. We evaluated whether an ${\mathbf{SSDE}}_{\mathbf{BMI}}$ can be determined from the patient’s body mass index ($\mathbf{BMI}$) with sufficient reliability in the case that a $\mathbf{SSDE}$ is not given by the CT scanner. For each of the three most used examination types, CT examinations of 50 female and 50 male patients were analyzed. The $\mathbf{SSDE}$ values automatically provided by the scanner were compared with ${\mathbf{SSDE}}_{\mathbf{BMI}}$ determined from $\mathbf{C}{\mathbf{TDI}}_{\mathbf{vol}}$ and $\mathbf{BMI}$. A good accordance of ${\mathbf{SSDE}}_{\mathbf{BMI}}$ and $\mathbf{SSDE}$ was found for the chest and abdominal regions. A low correlation was observed for the head region. The presented method is a simple and practically useful surrogate approach for the chest and abdominal regions but not for the head.

## INTRODUCTION

The volumetric computed tomography dose index ($\mathrm{C}{\mathrm{TDI}}_{\mathrm{vol}}$) and the associated dose length product ($\mathrm{DLP}$) are routine dosimetry metrics that are currently used to monitor radiation exposure to patients undergoing computed tomography (CT)^(1, 2)^. However, $\mathrm{C}{\mathrm{TDI}}_{\mathrm{vol}}$ is of limited value as it does not represent the amount of exposure an individual patient receives but only indicates the amount of radiation that a cylindrical polymethyl-methacrylate (PMMA) phantom with a diameter of either 16 cm or 32 cm would receive^(3–5)^. For this reason, the size-specific dose estimate ($\mathrm{SSDE}$) index was introduced, which incorporates the water-equivalent skin-to-skin diameter of the patient^(6, 7)^. This methodology is an IEC standard since 2019^(8)^.

The advantage of $\mathrm{SSDE}$ is that individual patient size is used to estimate the dose. This is an important feature because $\mathrm{C}{\mathrm{TDI}}_{\mathrm{vol}}$, which is independent of patient size and scan length, would indicate too low a dose for a selected examination in slim patients, while it would be the opposite in obese patients. Dose management systems are currently being established to report radiation exposure for further evaluation when diagnostic reference values are significantly exceeded without reason^(9)^. Improved estimates of the exposure are certainly necessary to automatically and reliably identify unjustified high doses of radiation. For the latter, $\mathrm{SSDE}$ seems well suited because it is patient size-specific.

However, the calculation of the $\mathrm{SSDE}$ may fail if the patient’s body contour cannot be clearly identified in the overview images or is outside of the overview images and the diameter in the center slice is therefore incorrectly determined or not determined at all. Although methods have been developed which recognize these problems and improve the calculations, they have not yet been implemented in the scanners currently in use^(10)^. In addition, the calculation of the diameter and thus the $\mathrm{SSDE}$ from all CT slices is time-consuming and only possible afterward^(11)^.

Therefore, it seems an interesting approach to estimate the $\mathrm{SSDE}$ without information about the water-equivalent diameter of the patient. Although other research groups have already addressed this issue, they have done so with different approaches, most of which did not differentiate by gender or include pediatric patients^(12–16)^. Several research groups already addressed the correlation of body mass index ($\mathrm{BMI}$) and water-equivalent diameter but considered only a specific body region, limited themselves to individual types of CT examination or compared results from different scanners.

In this study, we evaluate a simple approach for female and male patients using $\mathrm{BMI}$ to estimate the $\mathrm{SSDE}$. $\mathrm{BMI}$ is readily available and can often be retrieved from the hospital information system to estimate $\mathrm{SSDE}$ from $\mathrm{C}{\mathrm{TDI}}_{\mathrm{vol}}$ for the most frequently examined body regions of head, chest and abdomen. When CT scanners and dose management systems do not provide this value, this could be beneficial.

## MATERIALS AND METHODS

This retrospective study complies with the Declaration of Helsinki and was carried out with the approval of the Ethics Committee of the Friedrich Schiller University Jena. All patients provided written informed consent.

### Patient population

For each of head, chest and abdomen, 100 CT examinations were retrospectively selected from the hospital database for 50 female and 50 male adult patients (see Table 1). The sample size was chosen to obtain a reasonable statistical result with a reasonable amount of information collection. The biometric data for calculating the $\mathrm{BMI}$ were taken from the electronic health records and were not from the period of 2 weeks before to 2 weeks after the examination. None of the patients had any prosthesis or other implants within the scan field. The baseline patient data and the scan parameters are summarized in Table 1.

**Table 1:** Baseline patient data and scan parameters.

Head						
	Female (*n* = 50)		Male (*n* = 50)		Scan parameter	
	Mean ± SD	Range	Mean ± SD	Range	Tube voltage (kVp)	120
Age	66.4 ± 14.1	21–92	67 ± 15.5	28–88	Effective mAs (mean)	313
BMI	26.7 ± 5.9	15.6–44.2	26.4 ± 4.7	16.2–43.3	Detector coverage (mm)	80
CTDI	53.8 ± 6.6	40.9–78.9	52.9 ± 4.4	42.0–82.9	Table increment (mm)	80
SSDE (CT)	49.8 ± 5.6	40.3–73.4	48.2 ± 4.0	39.7–57.2	Scanmode	Step and shoot, axial
Chest						
	Female (*n* = 50)		Male (*n* = 50)		Scan parameter	
	Mean ± SD	Range	Mean ± SD	Range	Tube voltage (kVp)	120
Age	64.6 ± 12.4	36–89	65.1 ± 12.1	27–88	Effective mAs (mean)	96
BMI	26.8 ± 5.6	15.9–42.0	27.1 ± 4.0	20–38.4	Detector coverage (mm)	80
CTDI	5.0 ± 2.8	1.9–11.8	6.8 ± 2.4	2.8–12.9	Pitch factor	0.99
SSDE (CT)	5.1 ± 2.1	2.6–9.4	6.5 ± 1.7	3.3–9.6	Scanmode	Spiral
Abdomen						
	Female (*n* = 50)		Male (*n* = 50)		Scan parameter	
	Mean ± SD	Range	Mean ± SD	Range	Tube voltage (kVp)	120
Age	66.2 ± 12.0	33–90	66.1 ± 10.9	37–82	Effective mAs (mean)	109
BMI	24.4 ± 4.9	17.2–37.6	27.0 ± 5.6	17.8–39.9	Detector coverage (mm)	80
CTDI	6.4 ± 1.8	4.4–11.1	8.6 ± 3.2	4.5–16.8	Pitch factor	0.99
SSDE (CT)	7.2 ± 1.1	5.7–9.8	8.4 ± 1.8	5.8–12.7	Scanmode	Spiral

### Technical specification

All scans had been performed with the same CT scanner (Revolution CT, General Electric Company, USA), which automatically provides ${\mathrm{CTDI}}_{\mathrm{vol}}$ and $\mathrm{SSDE}$. The scanner is a 256 slice system and has a patient coverage in *z*-direction of up to 160 mm, resulting in 0.625 mm thickness per detector element.

$\mathrm{C}{\mathrm{TDI}}_{\mathrm{vol}}$ and $\mathrm{DLP}$ were determined by the scanner for patient-mimicking cylindrical PMMA phantoms with diameter of 16 and 32 cm for the head and body regions, respectively^(3)^. By contrast, the American Association of Physicists in Medicine (AAPM) Report 220 proposes to use the patient’s effective water-equivalent diameter, ${D}_{\mathrm{W}}$, which can be extracted from localizer images in lateral and anterior–posterior directions or from cross-sectional CT images to calculate $\mathrm{SSDE}$^(7)^. The scanner used in our work calculates ${D}_{\mathrm{W}}$ as

**Figure 1:**
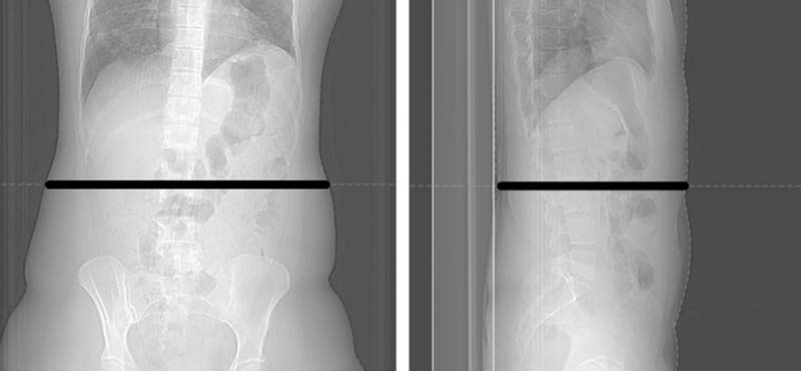
Localizer images for an abdominal scan; the black lines show the diameter of the patient in the central slice position; left: lateral direction ($\mathrm{LAT}$); right: anterior–posterior direction ($\mathrm{AP}$); the $\mathrm{SSDE}$ values output by the CT scanner used in this study are calculated using this center-slice method.

(1) \begin{equation*} {D}_{\mathrm{W}}=\sqrt{\mathrm{LAT}\bullet \mathrm{AP}} \end{equation*}

using the patient’s diameter in lateral direction, $\mathrm{LAT}$, and in anterior–posterior direction, $\mathrm{AP}$, as illustrated on a central slice of an exemplary scan region (center-slice method) in Figure 1.

In the AAPM Report 220, conversion factors, $f({D}_{\mathrm{W}})$, are available for both PMMA phantoms for values of ${D}_{\mathrm{W}}$ in the range from 8 to 45 cm to calculate $\mathrm{SSDE}$ from the $\mathrm{C}{\mathrm{TDI}}_{\mathrm{vol}}$ values output from the scanner:

(2) \begin{equation*} \mathrm{SSDE}={\mathrm{CTDI}}_{\mathrm{vol}}\bullet f\left({D}_{\mathrm{W}}\right) \end{equation*}

The conversion factors, $f({D}_{\mathrm{W}})$*,* are based on a curve fit between physical measurements and Monte Carlo simulations^(6, 7)^. However, in cases where ${D}_{\mathrm{W}}$ is not automatically calculated, we propose a simple method to derive $\mathrm{SSDE}$ from the patient’s $\mathrm{BMI}$ and the ${\mathrm{CTDI}}_{\mathrm{vol}}$ output from the scanner, where the metric, ${\mathrm{SSDE}}_{\mathrm{BMI}}$ (index $\mathrm{BMI}$ indicates calculation based on the patient’s individual $\mathrm{BMI}$) is calculated as follows:

(3) \begin{equation*} \mathrm{S}{\mathrm{SDE}}_{\mathrm{BMI}}={\mathrm{CTDI}}_{\mathrm{vol}}\bullet f\left(\mathrm{BMI}\right) \end{equation*}

The $\mathrm{BMI}$ was calculated according to the equation

(4) \begin{equation*} \mathrm{BMI}=\frac{\mathrm{Weight}\ }{{\mathrm{Height}}^2}. \end{equation*}

To determine the conversion factors $f({D}_{\mathrm{W}})$ used by our CT scanner, the ratios between the available $\mathrm{SSDE}$ and ${\mathrm{CTDI}}_{\mathrm{vol}}$ values were calculated for all patients in each anatomic group (i.e. head, chest, and abdomen):

(5) \begin{equation*} f\left({D}_{\mathrm{W}}\right)=\frac{\mathrm{SSDE}}{{\mathrm{CTDI}}_{\mathrm{vol}}} \end{equation*}

In addition to Report 220, the AAPM released Report 293 for head CT^(17)^. The conversion factor, $f({D}_{\mathrm{W}})$, is calculated there with the formula

(6) \begin{equation*} f\left({D}_{\mathrm{W}}\right)=1.9852\bullet{e}^{-0.0486\bullet{D}_{\mathrm{W}}} \end{equation*}

Most of the currently used scanners calculating the $\mathrm{SSDE}$ still according to AAPM Report 220 for the head section, so we decided to consider both approaches for the head section for this work.

A similar functional relationship to the $\mathrm{BMI}$ has now been examined. The approach should work if ${D}_{\mathrm{W}}$ and $\mathrm{BMI}$ are linearly related. This was initially checked visually as shown using a scatterplot. To distinguish, the conversion factor is now referred to as $f(\mathrm{BMI})$. To determine the functions for $f(\mathrm{BMI})$ for each group, the calculated gender-separated values $f({D}_{\mathrm{W}})$ (*n* = 50 each) were plotted against the corresponding individual $\mathrm{BMI}$ values and were then fitted to an exponential function of the form

(7) \begin{equation*} f\left(\mathrm{BMI}\right)=m\bullet{e}^{b\bullet \mathrm{BMI}} \end{equation*}

where $m$ and $b$ are fit parameters. MATLAB 2017a was used for these curve fits to extract the values of $m$ and $b$ for both genders and each target region (see Figure 4). With known parameters $m$ and $b$, the fit function can be used to later calculate ${\mathrm{SSDE}}_{\mathrm{BMI}}$ from $\mathrm{BMI}$ according to Eq. (3).

### Statistical analysis

To assess the validity of our approach, the ${\mathrm{SSDE}}_{\mathrm{BMI}}$ values were compared to the corresponding $SSDE$ values by calculating the coefficient of determination *R*^2^ of the linear fit through the point of origin. A coefficient of determination of ≥0.25 was classified as weak correlation, ≥0.5 as moderate and ≥0.75 as substantial correlation^(18)^.

In addition, Bland–Altman diagrams were created to show the variation in the results. For this purpose, the mean of two corresponding values ($\mathrm{SSDE}$ and ${\mathrm{SSDE}}_{\mathrm{BMI}}$) was plotted against the difference of the two values. For normally distributed differences, one would expect 95% of the differences to lie between two lines, calculated as

(8) \begin{equation*} \overline{d}\pm 1.96\bullet \mathrm{SD}\ (d) \end{equation*}

where $d$ is the difference between $\mathrm{SSDE}$ and ${\mathrm{SSDE}}_{\mathrm{BMI}}$, and $\overline{d}$ denotes the mean of all individual $d$ for each target region and gender. Almost all pairs of calculation of the two methods should ideally be closer to each other than these extreme values, which are called the ‘95% limits of agreement’^(19)^.

## RESULTS

**Figure 2:**
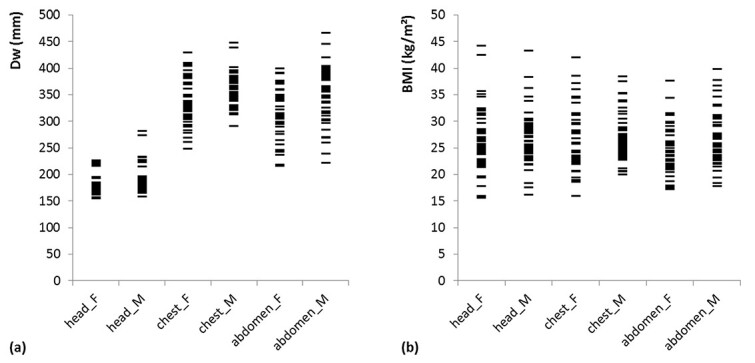
Diagrams showing (**a**) the distribution of ${D}_{\mathrm{W}}$ as calculated by the scanner and (**b**) the $\mathrm{BMI}$ for all patient groups; each small horizontal line segment represents the data of one patient.

Figure 2 shows the distributions of the water-equivalent diameter ${D}_{\mathrm{W}}$ determined by CT and $\mathrm{BMI}$ for all examinations. As expected, the values for ${D}_{\mathrm{W}}$ for the head are distinctly less scattered compared to the corresponding values for $\mathrm{BMI}$, indicating the low correlation between head diameter and $\mathrm{BMI}$.

**Figure 3:**
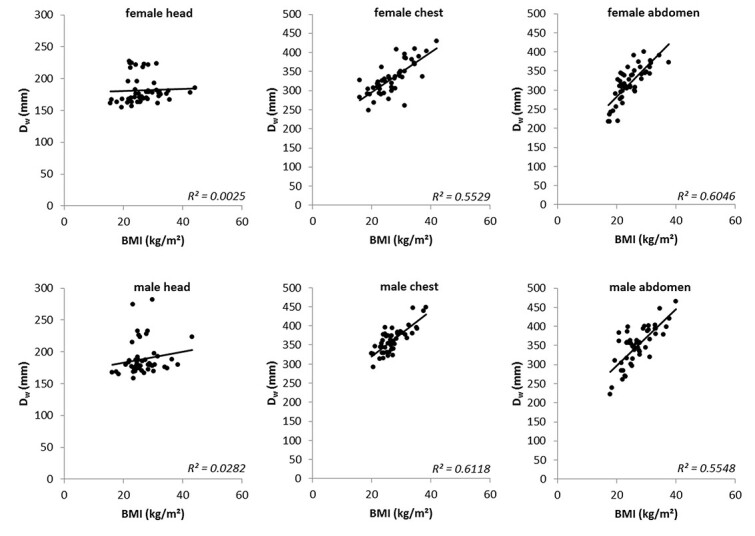
Scatterplots between ${D}_{\mathrm{W}}$ and $\mathrm{BMI}$ for all target regions and both genders; the black solid lines represent the linear fit; the goodness of that linear fit is given as *R*^2^ in the right edge of each diagram.

Figure 3 shows the scatterplots between ${D}_{\mathrm{W}}$ and $\mathrm{BMI}$ for the different body regions together with the linear regression lines. From these graphs, as well as from the *R*^2^ values of the linear fit functions, the poor correlation in the head region between ${D}_{\mathrm{W}}$ and $\mathrm{BMI}$ is clear. In order to show to what extent this affects the calculation of the $\mathrm{SSDE}$, we have nevertheless continued the evaluation for the head region. The linear correlation is moderate for both the thoracic and abdominal regions, with a slightly better fit for the abdominal region in female patients and for the thoracic region in male patients.

**Figure 4:**
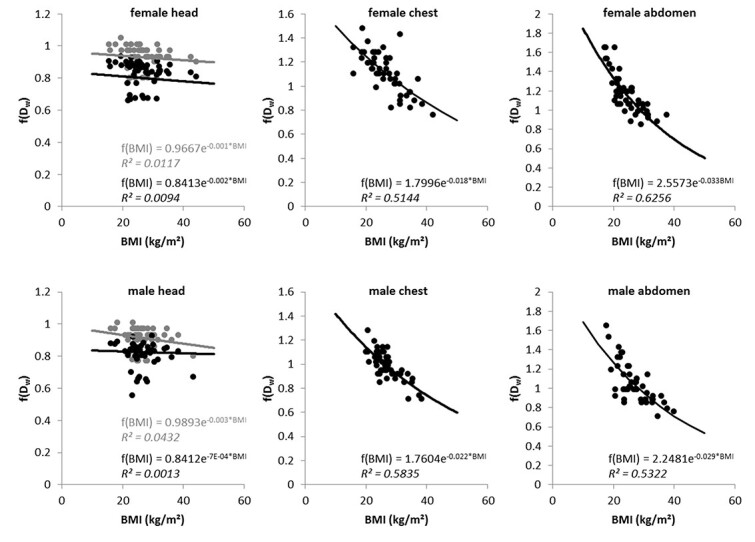
Scatterplots between $\mathrm{BMI}$ and $f({D}_{\mathrm{W}})$ for all target regions; the lines indicate the fit to the data; the formulas developed of these fits can be used to calculate $f(\mathrm{BMI})$ from the $\mathrm{BMI}$ data of the patients; for the diagrams of the head section, light gray is the data set calculated according to AAPM 220, black stand for calculations with conversion factors as described in AAPM Report 293.

Figure 4 shows the scatterplots between the conversion factors $f({D}_{\mathrm{W}})$ derived from Eq. (5) and the corresponding $\mathrm{BMI}$ for all patients and body regions together with the fit curves according to Eq. (7). The coefficients of determination *R*^2^ of the fit functions between $f({D}_{\mathrm{W}})$ and $\mathrm{BMI}$ are also given for each equation. All results for chest and abdomen can be classified as moderate correlation. The fit result is best for the abdominal region in females and is best for the chest region in males. For the head region, the results corroborate the expected missing correlation between the conversion factors $f({D}_{\mathrm{W}})$ and $\mathrm{BMI}$. The results for estimations according to the older AAPM Report 220 are reproduced in gray color. Comparing the *R*^2^, we see that the current recommendation of the AAPM to calculate the $f({D}_{\mathrm{W}})$ with the method described in Report 293 does not improve the results with our method. Although the use of *R*^2^ is not optimal for non-linear models, as it then often simulates better results, it is still a quick and easy way to get an initial overview by comparing data series^(20)^.

**Figure 5:**
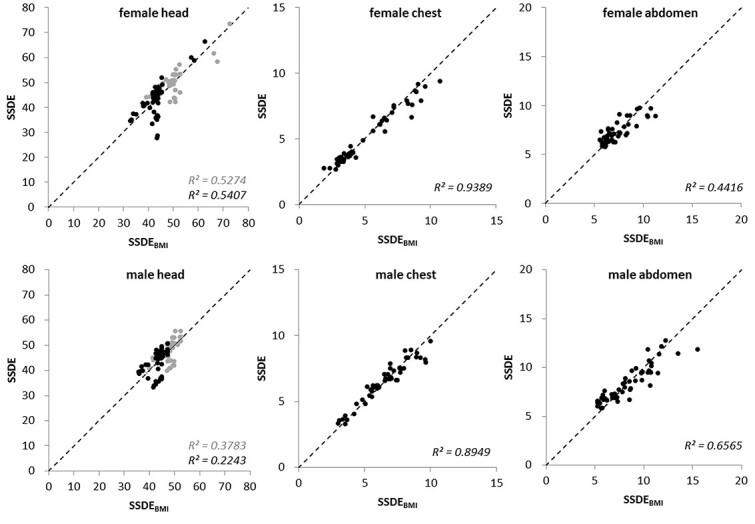
Scatterplots between ${\mathrm{SSDE}}_{\mathrm{BMI}}$ and $\mathrm{SSDE}$ for all three anatomic target regions and both gender; the black dashed lines represent the lines of identity; *R*^2^ stands for the determination coefficients between ${\mathrm{SSDE}}_{\mathrm{BMI}}$ and $\mathrm{SSDE}$; for the diagrams of the head section, light gray is the data set calculated according to AAPM 220, black stand for calculations with conversion factors as described in AAPM Report 293.

In Figure 5, the results for the calculated ${\mathrm{SSDE}}_{\mathrm{BMI}}$ are compared with the $\mathrm{SSDE}$. Correlations for the head region are again worst but can be classified as moderate. Although the previous findings of this study tend to suggest that the abdomen region might give the best results in the female group. Looking at *R*^2^ between ${\mathrm{SSDE}}_{\mathrm{BMI}}$ and $\mathrm{SSDE}$, it shows an substantial result in the chest region and only a weak correlation of both values for the abdominal region. The result of the assessment of the ${\mathrm{SSDE}}_{\mathrm{BMI}}$ for the male group is substantial in the chest region and is moderate for the abdominal region.

**Figure 6:**
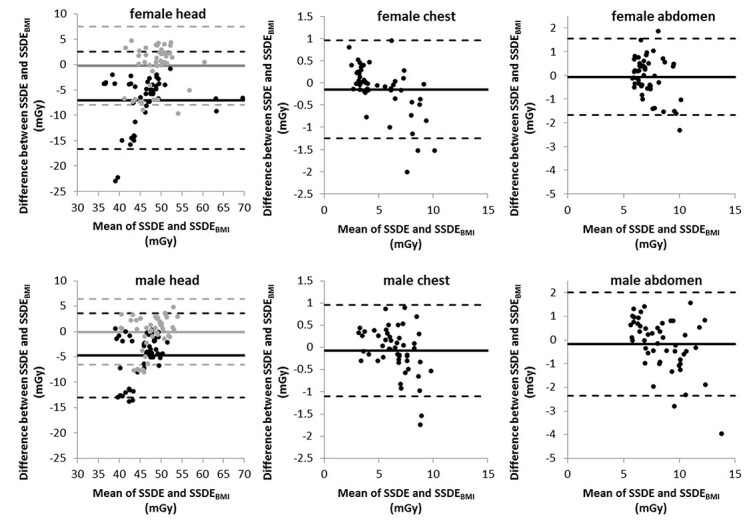
Bland–Altman-diagrams comparing $\mathrm{SSDE}$ and ${\mathrm{SSDE}}_{\mathrm{BMI}}$; the dots represent the result of a corresponding pair of $\mathrm{SSDE}$ and ${\mathrm{SSDE}}_{\mathrm{BMI}}$; the solid lines indicate the mean of the differences between corresponding $\mathrm{SSDE}$ and ${\mathrm{SSDE}}_{\mathrm{BMI}}$, and the dashed lines represent the mean of the difference plus or minus 1.96 multiplied with the standard deviation of the differences; for the diagrams of the head section, light gray is the data set calculated according to AAPM 220, black stand for calculations with conversion factors as described in AAPM Report 293.

As seen in Figure 6, 95% of the cases lie within the boundary lines (i.e. two to three values in this case) for all study groups. Thus, when assessing the results using this method, one can assume that an approximation of the $\mathrm{SSDE}$ with the help of the $\mathrm{BMI}$ is quite practicable even for the head region. In view of the previous results, however, this should be questioned critically.

## DISCUSSION

The proposed method is a quick and easy alternative to evaluate size-specific doses for patients with conspicuous ${\mathrm{CTDI}}_{\mathrm{vol}}$ values in situations where an automatically calculated $\mathrm{SSDE}$ is not provided by the CT scanner. It is not a substitute for an exact calculation, as recommended in the AAPM report, but can help physicians or radiographers to estimate the acceptability of an applied dose considering a reference level.

An advantage of our approach is that it uses the $\mathrm{BMI}$ in combination with derived equations for different body regions without the need to know the patient’s effective diameter. However, this approach is also limited because the $\mathrm{BMI}$ for a small person (child or small adult) could be the same as for a tall person, while the effective diameter and thus the exposed volume would most likely be significantly different. Parikh *et al.* investigated the extent to which body weight can also be used to determine $\mathrm{SSDE}$ in pediatric patients. The results differ depending on the patient’s body diameter^(5)^. Therefore, to improve the approximation of our proposed ${\mathrm{SSDE}}_{\mathrm{BMI}}$ to $\mathrm{SSDE}$, more patients and groups including pediatric patients need to be investigated in future studies^(21)^. Furthermore, the ratio of $\mathrm{BMI}$ to ${D}_{\mathrm{W}}$ can vary, depending on the proportions of muscle and fat tissue present and is age and gender effected^(22)^. These differences can also affect the correct estimation of the ${\mathrm{SSDE}}_{\mathrm{BMI}}$. Another uncertainty for the calculated $\mathrm{BMI}$ is possible weight loss, especially in oncology and intensive care patients, during the maximum allowable 2-week period between weight and height data collection and CT acquisition.

Considering only the Bland–Altman method to evaluate the results, one could come to the conclusion that the estimation of $\mathrm{SSDE}$ values for the head based on $\mathrm{BMI}$ is unproblematic. However, in comparison with the other results, we cannot recommend this. The lack of correlation between ${D}_{\mathrm{W}}$ and $\mathrm{BMI}$ in the area of the head is particularly evident in Figure 3. The coefficient of determination here is slightly better for men than for women, but both values are extremely poor and show that a calculation using our method cannot produce any useful values for ${\mathrm{SSDE}}_{\mathrm{BMI}}$ in the head region. There are recommendations to determine $\mathrm{SSDE}$ using specialized head scan band regardless of size and/or weight^(23)^. For the head region, the equations used in our study to calculate $f(\mathrm{BMI})$ show strikingly poor agreement with the values of $f({D}_{\mathrm{W}})$, which is reflected in the low values for *R*^2^ (see Figure 4). The changes in AAPM Report 293 compared to Report 220 have only a small effect on our findings. It appears that estimating $\mathrm{SSDE}$ values of the head region from a patient’s $\mathrm{BMI}$ is not accurate. As Figure 2 shows, the diameter of the head has only small variations in patients, although the $\mathrm{BMI}$ varies relatively strongly, resulting in weak or no correlation. This observation is also supported by a study by Alikhani *et al.*, who used an exponential relationship to calculate a size-specific ${f}_{\mathrm{size}}$ from $\mathrm{BMI}$ and assumed independence of both values for the head region^(12)^. Mehdipour *et al.* have investigated how $\mathrm{SSDE}$ and lateral body diameter correlate. The correlation for the abdominal and for the chest region they found are also seen in our investigations. However, they did not include gender in their study, where our results show a difference between female and male patients^(15)^. Xu *et al.* found that the $\mathrm{BMI}$ is better suited than body weight to estimate $\mathrm{SSDE}$. However, they calculated the $\mathrm{SSDE}$ from the patients water-equivalent diameter estimated from body weight and $\mathrm{BMI}$^(16)^. In our study, we directly estimated the ${\mathrm{SSDE}}_{\mathrm{BMI}}$ from the $\mathrm{BMI}$.

The results presented in Figure 5 show moderate correlations between ${\mathrm{SSDE}}_{\mathrm{BMI}}$ and $\mathrm{SSDE}$ for the abdominal region. The best agreement with a substantial correlation was observed for the chest region, suggesting that $\mathrm{BMI}$ for this region predicts $\mathrm{SSDE}$ well. The equations established with the scatterplots in Figure 4 provide feasible approaches for calculating $\mathrm{SSDE}$ in the chest and abdominal regions with almost similar accuracy as in the AAPM report. O’Neill *et al*. also investigated the applicability of $\mathrm{BMI}$ to estimate the individual patient radiation dose by calculating an effective diameter from the $\mathrm{BMI}$ and demonstrated a substantial correlation (*r* = 0.88) between these two values^(13)^. However, they only evaluated abdominal scans in 50 patients (female and male varied) in their study.

Discrepancies between ${\mathrm{SSDE}}_{\mathrm{BMI}}$ and $\mathrm{SSDE}$ may also be caused by the method implemented in the CT scanner to determine the effective diameter. The scanner used in our study determines the effective diameter from the central position of the scout images (see Figure 1), as described in the AAPM Task Group 204 report^(6)^. Because of its higher accuracy, the AAPM recommends calculating ${D}_{\mathrm{W}}$ using the slice-by-slice method from axial CT images^(7, 24, 25)^. However, the disadvantage of the latter method is the longer calculation time and that the results are obtained only retrospectively. In most cases, ${D}_{\mathrm{W}}$ calculated by the center-slice method is almost identical to the ${D}_{\mathrm{W}}$ calculated by the slice-by-slice method, although it has been shown that they can differ by up to 3.7% in abdominal phantom studies^(7)^. In patients, it could be even larger because the shape of the abdominal region varies more depending on weight, height and individual constitution. Boos *et al*. showed that the center-slice method results in a mean relative absolute error of 2–5% in pediatric and adult abdominal and chest CT compared with the slice-by-slice method^(14)^. For pediatric thoracic, abdominal and pelvic scans, Öszoykal *et al.* determined a root-mean-squared error of 1.2–11% when comparing the $\mathrm{SSDE}$ from the center-slice method with the slice-by-slice determination^(26)^. Furthermore, $\mathrm{SSDE}$ also depends on the positioning of the patient in the isocenter of the scanner gantry, which can lead to inaccuracies if performed incorrectly^(7, 27, 28)^. The IEC standard for $\mathrm{SSDE}$ cites an included neck in the scanned anatomy, an actual scan length that exceeds the range of the overview radiogram, unilateral or bilateral limb scans or foreign objects located in the scan field as possible causes of miscalculation of the $\mathrm{SSDE}$ by the scanner software^(8)^. These facts illustrate that the values for $\mathrm{SSDE}$ adopted as reference in our study also need to be reviewed and carefully evaluated.

In general, the use of $\mathrm{SSDE}$ is very helpful and diagnostic reference levels should be set to $\mathrm{SSDE}$ instead of ${\mathrm{CTDI}}_{\mathrm{vol}}$. Although the ${\mathrm{CTDI}}_{\mathrm{vol}}$ is a useful indicator of radiation dose, it does not represent the dose received by the patient due to the neglect of size. Therefore, in very slim or obese patients, the ${\mathrm{CTDI}}_{\mathrm{vol}}$ should be compared with the $\mathrm{SSDE}$ to check the plausibility of the ${\mathrm{CTDI}}_{\mathrm{vol}}$ value output by the scanner. For the optimization process of various CT examinations, the $\mathrm{SSDE}$ is an important variable for adapting the radiation exposure to as many body constitutions as possible. As described by Lyra *et al.*^(29)^, there is only a low correlation between the effective dose calculated with the $\mathrm{DLP}$ compared to the $\mathrm{SSDE}$. It can therefore be assumed that the value for the effective dose calculated from ${\mathrm{CTDI}}_{\mathrm{vol}}$ and $\mathrm{DLP}$ should also be critically examined, especially for patients in whom ${\mathrm{CTDI}}_{\mathrm{vol}}$ and $\mathrm{SSDE}$ differ greatly from one another.

## CONCLUSION

A patient’s $\mathrm{BMI}$ can be used in CT scans of the chest and the abdomen to efficiently estimate $\mathrm{SSDE.}$ This ${\mathrm{SSDE}}_{\mathrm{BMI}}$ has an almost similar accuracy to that derived from images. For CT imaging of the skull or brain, using $\mathrm{BMI}$ does not show good accuracy because the effective diameter of the head does not vary much in adults. When calculation of the $\mathrm{SSDE}$ from images is not possible, e.g. because body parts of the patient are not included in the image or localizer images, or because of missing software to calculate $\mathrm{SSDE}$, the proposed methodology of using $\mathrm{BMI}$ can be applied.
